# Primary anaplastic large cell lymphoma of the central nervous system in a child: A case report

**DOI:** 10.1097/MD.0000000000021115

**Published:** 2020-07-17

**Authors:** Shuo Feng, Qian Chen, Jinxiao Chen, Ping Zheng, Kangping Ma, Bojing Tan

**Affiliations:** Department of Neurology, Children's Hospital Affiliated to Capital Institute of Pediatrics, Beijing, China.

**Keywords:** anaplastic lymphoma kinase-1-positive, anaplastic large cell lymphoma, children, intracranial hypertension, primary central nervous system lymphoma

## Abstract

**Introduction:**

To report the clinical characteristics of primary central nervous system T-cell lymphoma with anaplastic lymphoma kinase-1 (ALK-1) positive in an 8-year-old male.

**Patient concerns:**

The patient presented cognitive impairment, dizziness, vomiting, fever, and convulsions during the disease, followed by progressive and persistent severe headache, progressive increase of intracranial pressure, indifference, disorder of consciousness, mild increase in white blood cells in cerebrospinal fluid, progressive decrease of sugar, progressive increase of protein, abnormal signal of left parietal-occipital, local meningeal enhancement, and cerebrospinal fluid cytology.

**Diagnosis:**

He was diagnosed with ALK-1-positive central nervous system T-cell lymphoma.

**Interventions:**

Meropenem and vancomycin were administered to counter the infection, while dexamethasone alleviated the inflammation.

**Outcomes:**

The patient died of cerebral hernia due to intracranial hypertension in the eighth week of the disease.

**Conclusions:**

PCNS ALK-1-positive anaplastic large cell lymphoma is extremely rare. Also, it is difficult to distinguish from central meningeal lymphoma and central nervous system infection, which might lead to delayed diagnosis. However, early diagnosis depends on the pathological diagnosis of brain tissue biopsy.

## 1 Introduction

Primary lymphoma in the central nervous system is rare, with an incidence of about 1/100,000.^[1]^ T-cell lymphoma accounts for <20% of all cases, which is also designated as a rare occurrence.^[2,3]^ Anaplastic large cell lymphoma (ALCL) is a type of T-cell lymphoma expressing CD30. The majority of the ALCLs are anaplastic lymphoma kinase (ALK)-positive, while a few are negative. Moreover, ALCL can be detected in any age group, primarily in males <50-years-old and adolescents.^[4]^ The main clinical manifestations include cranial hypertension, headache, epilepsy, disturbance of consciousness, memory loss, hemiplegia, and abnormal behavior. The lesions are mainly detected in the parietal lobe, frontal lobe, temporal lobe, and occipital lobe and can invade the pia mater.^[4,5]^ ALCL was first reported as ALK-1 positive pleomorphic or histiocytic-like lymphoma by Stein et al in 1985.^[6]^ According to the World Health Organization (WHO) classification criteria for the central nervous system lymphoma, ALCL is a specific subtype of T-cell lymphoma, accounting for 10% to 15% of children's non-Hodgkin lymphoma.^[7]^ The incidence of primary central nervous system (PCNS) ALCL was low because the number of males was more than that of females. The disease can occur in any age group. Currently, only a few reports have described this disease worldwide, especially in children. Herein, we reported a case of ALK-positive ALCL of the PCNS. The onset and progress of the disease differed from those reported previously. In this study, we summarized the characteristics, clinical progress, cerebrospinal fluid (CSF), and imaging of the cases to provide some clues for the diagnosis and treatment of suspected intracranial infection and intracranial hypertension.

## 2 Case presentation

Standard care is performed, so ethical approval is not applicable in this study. Written informed consent was obtained from the patient.

An 8-year-old male, who suffered from consciousness, dizziness, and vomiting for 3 weeks, fever for 3 days, and convulsion 1 time, was admitted to the local hospital. The child suffered from unconsciousness, dizziness, headache, and fever during the course of the disease. Convulsion was a generalized tonic-clonic seizure, which lasted for 30 minutes before relief. The patient was diagnosed as “viral encephalitis.” Acyclovir was given for the antiviral treatment and mannitol to reduce the intracranial pressure. However, the condition of the patient did not improve significantly. The consciousness gradually changed from vagueness to lethargy, accompanied by severe headaches. Hence, he was admitted to Capital Institute of Pediatrics, Beijing, China, for “central nervous system infection.’

Physical examination on admission: body temperature 37.9°C, heart rate 70 to 80 per minute, respiratory rate18 to 20 per minute. blood oxygen saturation 100%, blood pressure 133/79 mm Hg. Other manifestations included lethargy, apathy after awakening, positive neck resistance, Kernig sign was positive, Brudzinski sign was negative, Babinski reflex was positive bilaterally, other pathological signs were negative, and pyramidal tract sign was positive. Any specific abnormality was not detected in the cardiopulmonary abdominal examination.

**Figure 1:**
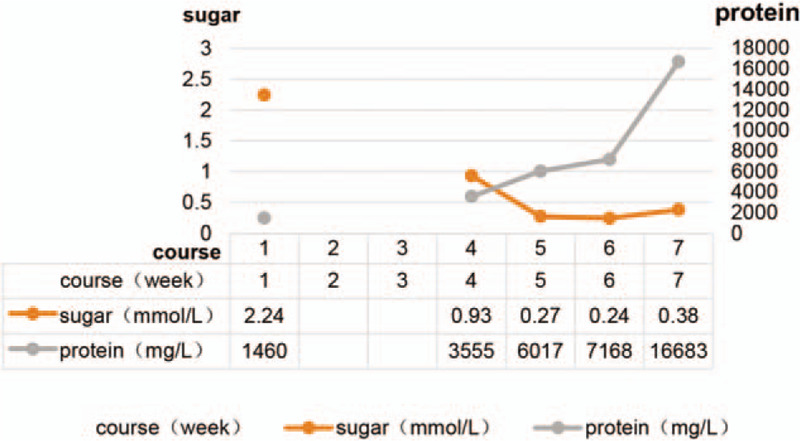
Altered trend of cerebrospinal fluid sugar and protein.

**Figure 2:**
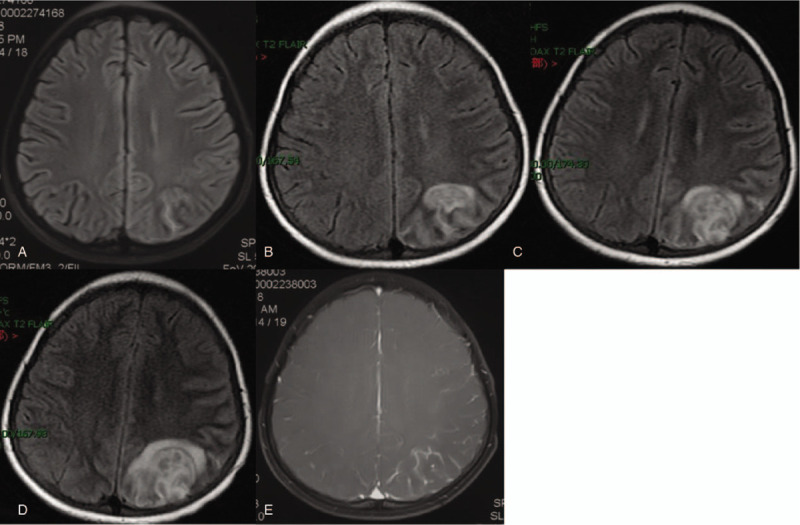
Changes in cranial magnetic resonance from week 1 to 6 during the disease. A, Abnormal signal of the left parietal lobe in the first week of onset; B, Left parietal lobe lesion enlarged in the fourth week of onset; C, Left parietal lobe lesion continued to enlarge and surrounded the edema in the fifth week of onset; D, The lesion area enlarged further, and the surrounding edema was obvious in the sixth week of onset; E, Local meningeal enhancement in the left parietal lobe.

Laboratory and radiological findings: Blood routine revealed that white blood cells count was16.2×10^9^/L, neutrophil count was 69%, and the proportion of lymphocytes was 28% and blood biochemistry and coagulation function were normal. The pressure of the CSF increased to >330 mmH_2_O, the white blood cell number of the CSF fluctuated from 24 to 300×10^6^/L, the sugar of the CSF decreased progressively (the lowest was 0.24 mmol/L), and the protein of CSF increased progressively (the highest was 16683 mg/L). The changes in the CSF are shown in Figure 1. Magnetic resonance imaging (MRI) showed that the left occipital lobe was swollen, and abnormally strengthened meninges were observed. With the progress of the disease, the scope of the focus gradually expanded (Fig. 2). Several times of CSF tests include smears, culture and metagenomic next-generation sequencing were negative. No obvious abnormality was detected in the pathological analysis of CSF cells in the fifth week of the disease. Conversely, medium-large lymphocytes were detected in the cytological pathology of CSF in the seventh week of the course of disease. Also, CD30 and CD45R0 were expressed, as assessed by immunohistochemistry, indicating malignant lesions. T cell receptor (TCR) B and TCRG gene rearrangement clones were detected in the TCR gene rearrangement of CSF. No tumor and lymphadenopathy were found on the chest and abdomen enhanced computed tomography scan, and no primitive immature cells were detected in the bone marrow and peripheral blood routine examination.

Treatment: After admission, the suspected central nervous system infection was given antiviral treatment of acyclovir, meropenem, vancomycin, amphotericin B, fluconazole, antituberculosis, and other anti-infective medication, successively. The results of the detection of intracranial hypertension, headache, disorder of consciousness, CSF, and progressive aggravation of intracranial lesions showed a worsening trend. To alleviate the high intracranial pressure and headache, lumbar cistern drainage was used twice. The failure of continuous drainage was due to CSF blockage within 24 to 48 hours. To further clarify the nature of the lesions, the pathological examination of the brain tissue was carried out.

**Figure 3:**
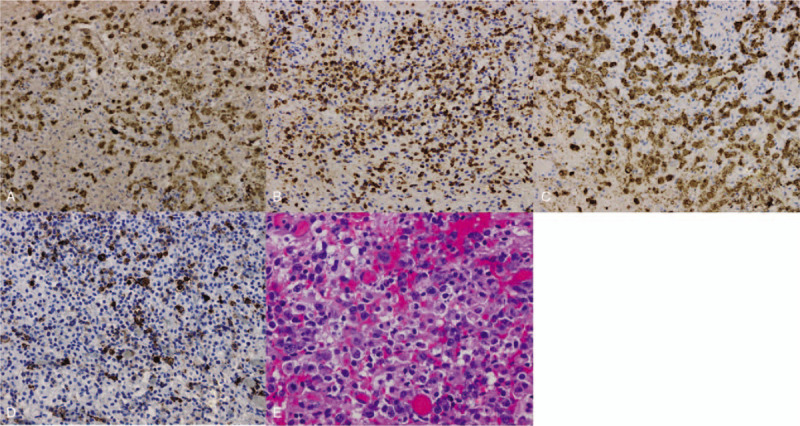
Pathological immunohistochemistry. A, Lymphoma cells ALK(+); B, Lymphoma cells CD3(+); C, Lymphoma cells CD30(+); D, Lymphoma cells EMA(+); E, Lymphoma cells with hematoxylin-eosin staining.

Pathological findings: Brain biopsy showed swelling of the brain tissue, disappearance of cerebral sulcus, thickening of cerebral surface veins, dark red color of the brain tissue, thickening of leptomeninges and arachnoid which were gray color, accompanied by capillary hyperplasia and scattered hemorrhage. In the necrotic brain tissue, heterotypic cells were found in diffused distribution, while in the wall of local blood vessels, heterotypic cells were found. Immunohistochemistry: GFAP (−), LCA (+), NSE (−), Ki-67 (about 50% in the hot spot area), langerin (−), S-100 (−), CD1 α (−), CD163 (histiocyte + +), CD3 (focus+), cd450ro (+), cxcl-13 (−), PD-1 (scattered +), CD21 (−), CD2 (+), CD5 (+), CD7 (+), CD4 (+), CD8 (+), CD30 (+), ALK (+), EMA (focus +), CD15 (−), CD79a (−), CD20 (−), non-Hodgkin lymphoma, T-cell-derived ALCL, ALK-positive, and pathological immunohistochemistry as shown in Figure 3.

Definite diagnosis: Primary ALCL with ALK-1-positive in CNS.

Prognosis: In the eighth week, the patient died of occipital hernia caused by intracranial hypertension.

## 3 Discussion and conclusions

Lymphoma can be divided into Hodgkin and non-Hodgkin lymphoma according to the pathological type. ALCL is a rare type of peripheral T-cell lymphoma, accounting for about 2% to 7% of non-Hodgkin lymphoma.^[8]^ On the other hand, while PCNSL is an extranodal malignant lymphoma originated from brain parenchyma, eye, meninges, spinal cord or spinal cord, which lacks the evidence of systemic lymphoma in diagnosis, accounting for 3% to 5% primary lymphoma in brain tumors. Strikingly, 95% of PCNSLs are B-cell lymphoma, while T-cell lymphoma constitutes the remaining 5%.^[9]^ The incidence rate of primary large cell lymphoma in the CNS is extremely low. Based on the fact that the incidence of primary ALCL in the CNS is extremely low, it could be deemed as a rare disease in children. Furthermore, due to the lack of evidence on systemic lymphoma, it is easily misdiagnosed in the clinic.

The common nervous system symptoms of primary ALCL in the CNS are intracranial hypertension, headache, seizures, disturbance of consciousness, memory loss, hemiplegia, and abnormal behavior. The lesions may also involve the cortex of parietal, frontal, temporal, and occipital lobes and invade the pia mater.^[4,5]^ PCNSL has no systemic lymphoma symptoms, only central nervous system symptoms, and hence, in clinical diagnosis and treatment, the first diagnosis department is neurology. In previous studies, about 83% of the cases were diagnosed with CNS infection for the first time.^[10]^ This case presented an 8-year-old boy with headache and persistent intracranial hypertension as the prominent manifestation. At the beginning of the disease, white blood cells in the CSF increased slightly. Cranial MRI revealed localized meningeal lesions and mild cortical and subcortical edema; these manifestations were diagnosed as “viral encephalitis.” With the progress of the disease, the white blood cell count of the CSF increased gradually, glucose decreased, and protein increased significantly. The lesion of cranial MRI was still confined to the parietal-occipital lobe, but the focus was obviously enlarged. Suppurative meningitis, cryptococcal meningitis, and tuberculous meningitis were suspected successively. The CSF was subjected to pathogenic culture, antibody detection, and metagenomic next-generation sequencing, but no positive results were obtained. A variety of anti-infective treatments for bacterial, viral, tuberculosis, and fungal infections were not effective. In the seventh week, the disease was diagnosed as primary ALCL of the CNS by brain biopsy.

At present, the standard clinical methods for the diagnosis and monitoring of central nervous system tumors have some limitations. The most common noninvasive method is neuroimaging. MRI displays tumor progression with a sensitivity of 73.3%. The most common site of PCNSL is the cerebral hemisphere (31%), and other common sites are the corpus callosum (16%), basal ganglia and thalamus (16%).^[11]^ The typical PCNSL features are T1-weighted low or equal signal intensity, T2-weighted equal or high signal intensity, diffusion limitation, meningeal involvement, and focal enhancement. PCNSL is not specific. In adults, leptomeninges are often involved in metaplastic large cell lymphoma in the central nervous system,^[12]^ while in children, the focus and pia mater are characterized by inhomogeneous enhancement. Thus, in children, central nervous system infections are diagnosed before the biopsy, which is the characteristic of PCNSLs in children.^[13–16]^ In this case, cranial contrast-enhanced MRI showed abnormal enhancement of leptomeninges and high signal intensity of brain parenchyma T2-weighted lesions, which was in agreement with the imaging features of PCNSL, but difficult to distinguish from the central nervous system infection before the biopsy. Therefore, for pediatric cases, when the diagnosis of central nervous system infection is planned but the anti-infective treatment is ineffective, the possibility of primary lymphoma in the CNS should not be excluded.

Intriguingly, CSF circulates rapidly in the cerebral ventricle and spinal cistern, can contact the tumor cells of the CNS, and carry the cells or nucleic acids released by the tumor.^[17]^ The pathological specificity of the CSF only appeared in the cases of invasion of leptomeninges, and the positive rate was relatively low.^[18]^ The child underwent cytopathological examination of CSF 2 times during the disease, and no abnormal lymphocytes were found in the fifth week of the course of the disease. The positive and specific results were obtained at the seventh week of the course of the disease, suggesting that the cases with leptomeningeal involvement should undergo cytological examination of CSF repeatedly; only one negative result cannot exclude the disease.

Furthermore, 50% to 75% of the cases were positive for ALCL ALK, while a few were negative. The prognosis of ALK-1-positive cases was better than that of ALK-1-negative cases.^[19]^ However, in primary ALCL of the CNS, the survival time of ALK-1-positive patients varies greatly. The shortest survival time is only 1 month, and the longest is several years;^[18]^ the mortality rate is about 50%. The causes of death are tumor metastasis, multiple organ failure, and intracranial hypertension.^[15,20]^ Intracranial hypertension is observed in about 1/3rd of PCNSL patients.^[21]^ In children, the proportion of intracranial hypertension is similar to that in adults.^[10]^ Previously, 4 cases of PCNSL with intracranial hypertension were reported, of which, 1 deceased due to increased intracranial pressure. Although continuous CSF drainage in the lumbar cistern can be relieved temporarily, the viscous CSF leads to poor drainage, and the patient died of cerebral hernia in 8 weeks. It is speculated that the prognosis of PCNSL children with intracranial hypertension is poor and the mortality is high. Previous studies reported that the increase in CSF protein level was positively correlated with adverse outcome,^[22]^ which was consistent with the present case. The CSF protein level of this child increased progressively, with a maximum of 16683 mg/L, showing a jelly-like appearance. Cerebral hernia caused by increased intracranial pressure due to poor circulation of CSF is considered to be the mechanism underlying poor prognosis of PCNSL.

In summary, children with primary and central nervous system large cell lymphoma are rare and could be easily misdiagnosed as central nervous system infections. The suspected cases of central nervous system infection with insufficient etiological evidence and poor anti-infection effect should be diagnosed by repeated CSF cytological pathology. Also, brain tissue biopsy should be performed as soon as possible in order to strive for the opportunity of treatment.

## Acknowledgments

We thank the support of our Clinical laboratory team for the pathogenic detection of cerebrospinal fluid.

## Author contributions

**Conceptualization:** Shuo Feng, Jinxiao Chen.

**Data curation:** Shuo Feng.

**Formal analysis:** Shuo Feng.

**Funding acquisition:** Ping Zheng.

**Investigation:** Shuo Feng, Qian Chen

**Methodology:** Jinxiao Chen.

**Resources:** Jinxiao Chen, Kangping Ma, Ping Zheng.

**Writing – original draft:** Bojing Tan, Kangping Ma.

**Writing – review & editing:** Bojing Tan, Qian Chen.
